# A harbor seal (*Phoca vitulina*) shows extensive respiratory control in sound production

**DOI:** 10.1186/s12862-025-02404-9

**Published:** 2025-09-02

**Authors:** Diandra Duengen, Yannick Jadoul, Andrea Ravignani

**Affiliations:** 1 Comparative Bioacoustics Research Group, Max Planck Institute for Psycholinguistics, Nijmegen, The Netherlands; 2 Zoo Cleves (“Tiergarten Kleve”), Kleve, Germany; 3 Center for Music in the Brain, Department of Clinical Medicine, Aarhus University, The Royal Academy of Music, Aarhus, Denmark; 4 Department of Human Neurosciences, Sapienza University of Rome, Rome, Italy; 5 Research Center of Neuroscience “CRiN-Daniel Bovet”, Sapienza University of Rome, Rome, Italy

**Keywords:** Bioacoustics, Respiratory production learning, Breathing control, Vocal learning, Animal training

## Abstract

The duration of animal vocalizations varies between and within species. Which mammals can learn to control this duration? Such respiratory production learning is a scarcely studied subcomponent of vocal learning. Here, we test the hypothesis that harbor seals (*Phoca vitulina*) are capable of respiratory production learning by testing whether a harbor seal can be trained to i) actively control its vocalization’s duration in two directions (short and long), and ii) exceed the pre-experimental vocalization’s duration (min = 0.202 s, max = 2.621 s). The seal learned to produce uninterrupted vocalizations spanning more than two orders of magnitude in duration, from 79 ms to 9.23 s. Our findings demonstrate a remarkable level of respiratory control in a harbor seal: this respiratory production learning encompasses an extensive range of sound durations and arises at a young age. Producing durations that span such a magnitude is hardly reported in the non-human animal literature; this capacity may be orthogonal to other vocal learning modules and should be tested in more species, both vocal production learners and non-learners.

**Supplementary Information:**

The online version contains supplementary material available at 10.1186/s12862-025-02404-9.

## Background

Some species can learn to produce new vocalizations, while others exhibit a set of innate vocalizations that minimally change over time. Vocal production learning is the ability to learn new vocalizations or modify existing ones based on experience [34]. A typical example of such vocal learning species are songbirds and parrots [6, 48]. Mammalian examples include certain species of bats [39, 41], cetaceans [13, 20], elephants [60], and seals [14, 51, 58, 59]. Crucially, most vocal learning literature focuses on the acquisition of novel call types [13] or the convergence of vocalizations towards sound models [20]; instead, vocal learning through non-imitative mechanisms [10] or the modification of single vocal parameters [58] remain largely unexplored.

The production of most mammalian sounds involves three components: respiration, phonation, and filter [19, 27, 62]. Such sound production emerges through respiration, where air serves as a “medium” and both the pulmonary apparatus (e.g., lungs, respiratory muscles, diaphragm) and passive recoil forces generate subglottal pressure– the air pressure below the vocal folds necessary for sound production [23, 29]. As the air passes through the larynx, the laryngeal tissue begins to vibrate, which introduces flow variation into the expiratory airstream. The resulting pressure disturbances generate a sound with different degrees of periodicity [44], but typically containing a fundamental frequency (f_0_; phonation) and harmonics. The airflow is then filtered through the supra-laryngeal vocal tract (filter), which generates formant frequencies [22].

The respiratory, phonatory, and filter components entail a complex mapping of interacting mechanisms [24, 26, 42, 47, 50, 53]. However, while phonation and filter typically shape the spectral properties of a sound, respiration and glottal resistance mainly influence its duration and amplitude [33]. Many mammals, including humans, modify single features, such as f_0_ [41], formants [25, 58] or duration [43], which can encode information when communicating.

As for the general case of sound production, vocal production learning spans three levels: respiratory, phonatory, and filter production learning, all involving a different level of control over their corresponding apparatus [34, 49]. Respiratory production learning is suggested to have preceded filter and phonatory production learning [34], and temporal vocal parameters are easier to adjust than spectral parameters [21, 34]. Respiratory production learning requires basic control of the respiratory system (and vocal fold adduction for the case of voiced sounds); altering frequency parameters demands close coordination between the respiratory and phonatory systems [34]. For example, human speech production requires solid respiratory control [43].

Respiratory production learning occurs in species that are vocal production learners and non-learners [46, 61]; hence respiratory production learning could be found in more species than those showing filter or phonatory production learning. Consequently, investigating such learning provides a comparative insight into vocal learning. Durational variation can emerge as a by-product of other vocal adaptations. When trained to vocalize above a certain frequency threshold, lesser spear-nosed bats (*Phyllostomus discolor*) modified not only the spectral parameters of their calls but also the calls’ duration and amplitude [40]. Conversely, in a similar experiment, harbor seals (*Phoca vitulina*) did not modify the duration of their calls [63]. Indirect evidence of respiratory production learning from durational adjustments may be confounded by emphasis on other acoustic parameters. So, how common is respiratory production learning across species?

Harbor seals are an ideal species to address this and related questions. They show some vocal learning capabilities [14, 17, 51, 55], with some control over the filter and phonatory level during sound production [25, 51, 63]. However, control over the third component– breathing– remains unexplored. This gap is intriguing, as breathing control in sound production may build upon seals’ physiological diving adaptations [4, 12]. As part of their adaptation to diving, harbor seals have excellent respiratory control. From a very young age, they can hold their breath while diving for extended periods [36, 37]. This breathing control facilitates closing off the airways [1], i.e., controlling air leaving or entering the lungs. Phocid seals typically exhale before diving which reduces buoyancy [54]. Interestingly, phocid lungs are not very big but have comparably large oxygen storage capacities [8, 9]. Despite their remarkable diving and breathing physiology, it is unknown whether harbor seals exert their fine-tuned respiratory control during sound production.

Does the harbor seal, a species with elaborate breathing control and possible phonatory and filter production learning, show respiratory production learning? Here, we test if a harbor seal can control the duration of its vocalizations in two separate directions (short and long), thereby probing respiratory production learning.

## Material & methods

### Aim of the study

To test respiratory production learning, we trained a juvenile male harbor seal to produce short and long vocalizations when presented with a discriminative stimulus (S_D_). We first trained the association between two arm gestures and short vs. long vocalizations. We then shaped the vocalizations’ durations in a series of training sessions. Finally, the seal’s ability for respiratory production learning was tested in a controlled experiment.

### Experimental setup

**Fig. 1:**
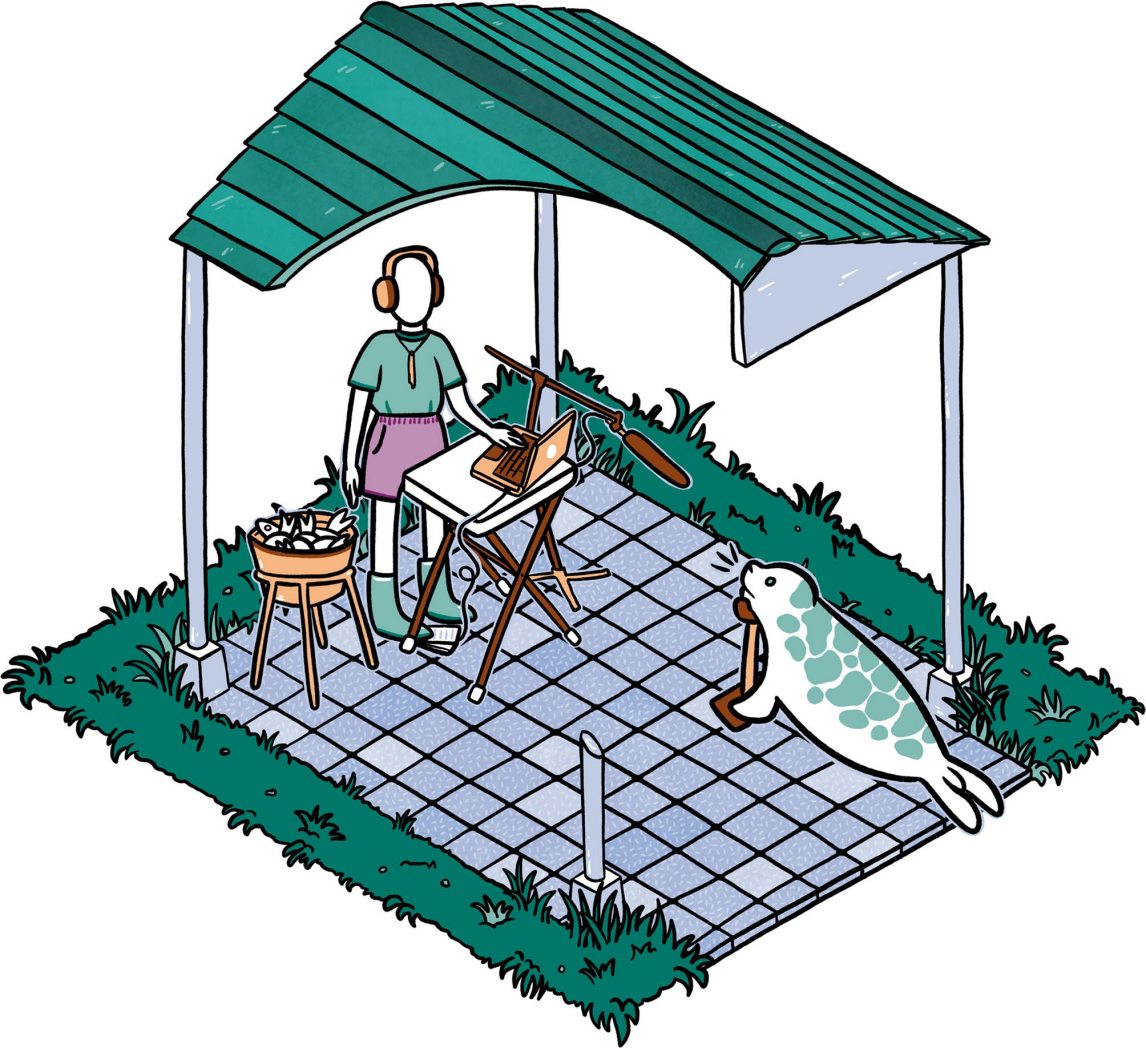
The seal is positioned on its station in front of the microphone and recorder, which records the seal’s vocal output. The experimenter stands in front of the laptop on which our custom-made application runs to assess the recorded response (see Fig. S1) of the seal

A one-and-a-half-year-old male harbor seal participated in the study. This young male was housed with four conspecifics in a 230,000-L freshwater tank and 300 m^2^ research enclosure at Zoo Cleves, Germany. We chose this animal to test respiratory production learning, while two other conspecifics, which also produced in-air vocalizations [15, 17], were tested in different tasks. All experiments were conducted on land in a research enclosure, neighboring the main enclosure. This research enclosure featured a 3 × 5 × 2.3 m carport above a 13.2 m^2^ paved platform (see Fig. 1). Audio recordings were collected with a Zoom H6 digital recorder connected to a Sennheiser ME-67 unidirectional microphone (frequency response: 40–20.000 Hz ± 2.5 dB, sampling rate: 48 kHz) covered by a foam windshield. The microphone pointed straight towards the seal at one meter.

### Training and testing procedure

We trained the seal through operant conditioning, reinforcing correct behavior positively with pre-cut pieces of fish. Incorrect behavior resulted in a three-second timeout. Initially, we trained the association of different movements of the right arm (discriminative stimulus, S_D_) with different durations. Concretely, a leftward movement (i.e., towards the body) was trained to elicit a short vocalization, while a rightward movement (i.e., away from the body) elicited a long vocalization. The experiment was conducted between September 2022 and January 2023– outside of the breeding season of harbor seals– and featured three phases.

First, the vocalization’s durations were *subjectively* assessed by the trainer. If the trainer presented the S_D_ for “short”, the seal was exclusively rewarded for a vocalization perceived as shorter than usual. Analogously, when the trainer presented the S_D_ for “long” the seal was exclusively rewarded for a longer vocalization. The seal participated in 21 sessions (on average 26.5 trials/session).

Next, to more *objectively* shape shorter and longer vocalizations, the vocalization’s duration was directly displayed to the experimenter via a custom-made, Python-based computer application (integrating acoustic analyses through Parselmouth; [5, 31, 32]). For this, the experimenter pushed a foot pedal to start real-time sound analysis and released the pedal to stop it. The seal participated in 14 sessions (average 29.5 trials/session). Once the seal responded to each S_D_ with a vocalization, testing started.

The third phase consisted of the actual experiment, where we tested the seal’s ability to adjust the duration of its vocalizations in alternating sessions, i.e., each experimental session consisted of only short or long trials, referred to below as sessions_short_ and sessions_long_, respectively. Initially, the seal was asked to produce shorter and longer vocalizations than a pre-determined duration threshold of 0.611 s. Crucially, the seal had to respond with short or long vocalizations upon presentation of the S_D_ for “short” and “long”. In other words, any vocalization below 0.611 s was correct in a session_short_ and any vocalization above 0.611 s was correct in a session_long_. This initial threshold of 0.611 s equaled the seal’s pre-experimental median duration (based on a subsample of every 7th pre-experimental vocalization). A response was counted as correct if its measured duration surpassed a session’s threshold (i.e., was shorter or longer than the threshold in a respectively short or long session). The threshold was adjusted once the seal reached the learning criterion: 80% correct choices in two consecutive sessions. The new threshold was then determined by calculating the median value of all responses in those last two sessions. For example, once the seal exceeded the duration of 0.611 s in at least 80% of all responses in two consecutive sessions_long_, the median duration of the responses in the successful sessions (e.g., 0.768 s) was calculated and served as a new threshold. In the subsequent sessions_long_ the seal was only rewarded for vocalizations longer than this new threshold until the learning criterion was reached again. The seal had a total of 110 sessions, each with 20 valid trials.

All responses were semi-automatically assessed through our application (Fig. S2). To start a trial, the experimenter pushed a foot pedal, presented the S_D_, and released it once the seal had finished vocalizing. This indicated a rough time window in the recording around the seal’s vocalization; based on this estimate, the application detected the vocalization, measured its duration, and compared it to the session’s threshold (see “Automatic Detection of Vocal Responses” in the Supplement). As an additional fail-safe mechanism, the application presented the spectrogram of the recording and its corresponding measurement to the experimenter. If the measured duration surpassed the threshold, the assessment was marked as correct; otherwise, the trial was marked as incorrect. Background noise or other external disruptions, causing a false measurement, triggered an invalid trial which was repeated right away.

### Acoustic analysis

The custom software analyzed the recorded vocalizations in real time and displayed an annotated spectrogram (Fig. S2). During the training phase, the application presented the spectrogram and measured the duration to help the experimenter decide which vocalizations to reinforce. During testing, the application additionally compared the measurement of each trial’s vocalization to the session’s pre-set threshold and proposed whether the trial was correct or incorrect. The experimenter then had three options: i) accept the application’s decision, ii) reject the application’s decision, or iii) repeat the trial. After the experiment, all durations were manually extracted to compare them to the application’s automatic measurements.

### Pre-experimental vocal repertoire

The pre-experimental repertoire comprises all vocalizations recorded before this experiment started (September 2022– November 2022), encompassing two vocalization types. These were pre-trained as part of the daily training routine. Concretely, the seal was trained to produce any type of sound and was directly rewarded whenever it did. This led to the emergence of vocal behavior under stimulus control (for more information on this type of procedure, see [17]). For this study, one of the two trained vocalizations was selected and trained to be produced upon presentation of the S_D_ (Fig. S1). The vocalization’s pre-experimental duration ranged from 0.202 s to 2.621 s (median: 0.611 s, Tab. S1).

### Statistical analyses

The experiment resulted in three different datasets: one dataset before the experiment– the pre-experimental vocalizations’ durations– and two datasets of the experimental data– the automatically measured durations of vocal responses and manually measured durations, excluding occasional trials with more than one recorded response. To test whether the seal successfully changed the duration of its vocalizations, we statistically compared the pre-experimental vocalizations to the manually annotated experimental vocalizations using a Mann–Whitney U test. Similarly, we statistically tested the change in duration between sessions with different thresholds. We tested the validity of the automatically measured durations by correlating them (Spearman's rank correlation coefficient) with the manually annotated vocal responses (see Fig. S3).

## Results

### Modification of duration throughout the experiment

**Fig. 2:**
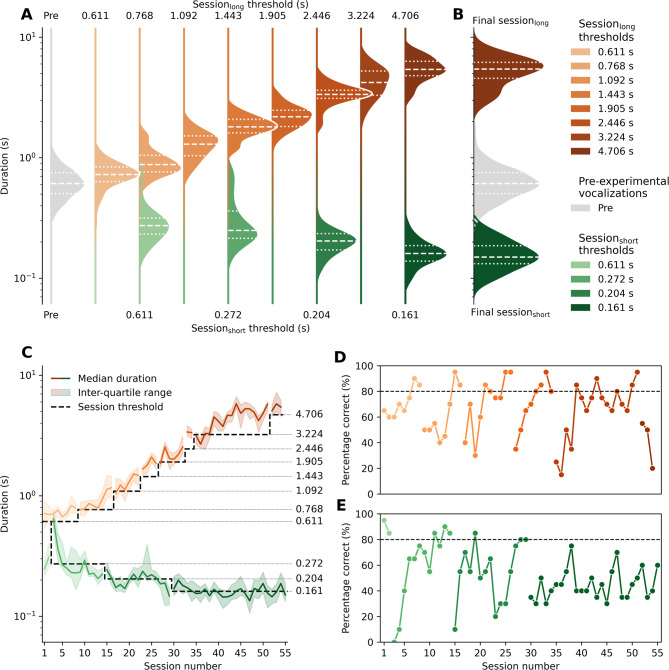
The experimental results show that the seal successfully learned to gradually increase and decrease the duration of its vocalizations. **A**) Grouped by session threshold, the sessions_long_ durations (orange) increase between successive thresholds, and vice versa for sessions_short_ (green). These distributions become increasingly distinct from the pre-experimental vocalizations’ durations (grey). The dashed lines indicate a distribution’s median, the dotted lines the first and third quartiles. Note the logarithmic scaling of the y-axis, spanning over two orders of magnitude. **B**) At the end of the experiment, there is virtually no overlap between the final session_short_ and session_long_ distributions and the pre-experimental one. **C**) Similarly, the median and inter-quartile range of the responses per session display the diverging vocal durations. **D**) The learning curve of the sessions_long_ shows the seal’s respiratory production learning progress over time. **E**) The learning curve of the sessions_short_ demonstrates the seemingly higher difficulty in consistently producing shorter vocalizations as the threshold lowers, especially for the final threshold of 0.161 s

Before the experiment, the duration of the seal’s vocalizations was 0.652 s ± 0.243 s (see Tab. S1). Over time, the seal learned to adjust its vocalizations to have an increasingly shorter or longer duration (Fig. 2). Durations in the final session_long_ (average: 5.336 s ± 1.312 s) significantly differed from the pre-experimental durations (Mann–Whitney U test, U = 0.0, *p* < 0.0001). Durations in the final session_short_ (average: 0.160 s ± 0.042 s) also significantly differed from the pre-experimental durations (Mann–Whitney U test, U = 15,443.0, *p* < 0.0001). Grouping the responses by their session’s threshold reveals significant changes in duration between every pair of consecutive thresholds (except between session_short_ thresholds 0.611 s and 0.272 s). Even the response durations measured at the start of the testing phase (session_short_ and session_long_ threshold 0.611 s) already significantly differ from the pre-experimental durations (see Fig. 2A and Table S1).

### Learning curves

The seal learned to respond to two different S_D_s with the same vocalization of different durations (short and long): when shown the S_D_ for “short”, the seal emitted a short vocalization, and when shown the S_D_ for “long”, a long vocalization. The seal learned to gradually decrease (short) or increase (long) the duration of its vocalization. This was achieved in 9.7 ± 5.6 sessions_short_ and 7.3 ± 4.4 sessions_long_ per threshold (Fig. 2D).

## Discussion

The harbor seal successfully learned to increase and decrease the duration of a selected vocalization. By the end of the experiment, its shortest and longest produced vocalizations differed by over two orders of magnitude, demonstrating an exceptional level of breathing control during vocal production (see below for a discussion on the role of glottal adduction). The pre-experimental durational range (minimum: 0.20 s; maximum: 2.62 s) was extended to a minimum and maximum duration of 0.079 s and 9.23 s, respectively. These results make a clear case of respiratory production learning in a harbor seal.

Overall, the seal reached each intermediate learning criterion (two sessions with ≥ 80% correct responses) rather quickly. On average, the seal took 9.7 ± 5.6 sessions_short_ and 7.3 ± 4.4 sessions_long_. The last two thresholds (0.161 s for “short”, and 4.706 s for “long”) were not learned before the experiment ended. Perhaps, the observed 0.079 s are the minimum duration the seal can reach. We noted that the vocalizations dropped in intensity as they got shorter. These short durations may be difficult to detect acoustically with our experimental setup, which may explain the comparably high number (*n *= 26) of sessions_short_. We suggest investigating such matters in an acoustic chamber to circumvent such limitations. While the last session_short_ threshold (0.161 s) took longer to reach than usual, we don’t see any indication of that within the final session_long_ results (4.706 s). On the contrary, with only four sessions completed, the seal produced some very long vocalizations. With more sessions, the seal would have likely produced even longer vocalizations. This is supported by the many values exceeding the last threshold, most notably a maximum duration of 9.228 s and the average number of sessions_long_ (7.6 ± 4.5, see above). However, since the experiment ended earlier– for reasons out of our control– the seal may not have had the opportunity to reach its maximally possible vocal duration. 

Some primates, carnivores and bats show respiratory control [40, 46, 49, 61]. Our experiment finds a drastically larger change in durational range than observed, for instance, in lesser spear-nosed bats [40]. In that study, one bat showed an altered median duration range of 0.054 s, compared to the pre-experimental range of 0.04 s. The experiment, however, did not aim to shorten or lengthen the bats’ vocalizations; such changes occurred as a by-product of another task, and likely underestimate the actual respiratory control in these bats. In contrast, Sutton et al. [61] directly tested whether (*Macaca mulatta*) could increase call duration and found that they did so up to three times the pre-experimental value (inferred from Fig. 3 in [61]). The harbor seal reported here demonstrated a durational increase of almost nine times on average, and a decrease of almost four, within just five weeks. Compared to previous work, we show specific control over the onset and offset of sound duration. Crucially, the seal learned to push the extent of one vocalization into two separate directions. It produced a range of vocal durations spanning over two orders of magnitudes. This range strongly exceeds the mammalian examples above [40, 61]. Overall, this indicates exceptional breathing control of in air sound production in a harbor seal.

Our results are relevant in the context of the evolution of speech: vocal learning is a crucial building block of speech. Respiratory production learning has been proposed as an evolutionary early form of vocal production learning and an instance of usage learning, as it primarily involves controlling the onset and offset of vocalizations [35], which might place this ability as an intermediate or transitional form between contextual learning and production learning [7]. The respiratory production learning we find in a mammal with a vocal apparatus remarkably similar to ours [52] may point to shared advanced respiratory control, perhaps convergently evolved in humans. Such control is essential to produce diverse speech sounds in humans [56] and to manage the timing of calls in harbor seal pups [2]. Our findings add additional evidence that harbor seals possess this capacity and can exert an extensive level of control over their vocalizations’ duration.

Our results also demonstrate that a harbor seal can learn to produce vocalizations of longer and shorter durations through a non-imitative experimental approach, providing an alternative to the classical vocal imitation studies [58, 59]. Past studies found vocal flexibility and indications of vocal production learning in harbor seals [51, 63]. Given the lack of conclusive evidence, this species’ ability and extent of vocal production learning is still debated [11]. Here, we have shown that a harbor seal can be conditioned to show novel temporal vocal features, thereby demonstrating respiratory production learning.

Our findings make it evident that the harbor seal was able to control the duration of its vocalizations. However, the specific mechanisms underlying sound production in this case remain unclear. Typically, the duration of a vocalization does not depend on the pulmonary apparatus alone but also involves the larynx [47, 57]. There, the duration of phonation (i.e., the production of a voiced sound) depends mainly on the phonatory lung volume (the amount of air that is available to produce a voiced sound) and the air flow rate (the moved air volume over time; [3]). The phonatory lung volume is restricted to vital capacity, i.e., the maximum amount of air that can be exhaled after having inhaled a maximum amount of air. The airflow rate is influenced through subglottal pressure, vocal fold adduction, and the resulting glottal adduction [27]. Which roles these physiological parameters played in our sound production experiment are unfortunately unclear. The seal vocalized with its nostrils open and mouth closed; the resulting sounds appeared to be voiceless (see Fig. S1). This suggests that the sounds were generated through nasal airflow rather than laryngeal phonation. Due to a lack of direct measurements (e.g., spirometry, airflow analysis), we cannot definitively determine the roles of the nose, larynx, or supra-laryngeal vocal tract in the production of these sounds. Future research should identify such roles, and include measurements of vital capacity and airflow rate [18], as well as directly investigate laryngeal involvement [28, 64] to clarify the contributions of different sound-producing structures in harbor seals. Future research should continue probing the limits of pinnipeds’ vocal production learning, ideally on the phonatory and filter level. Like our approach, one could test whether stepwise increments of vocal parameter values can lead to a different post-experimental parameter distribution, providing clear indications of vocal learning on these levels. Future research should include a bigger sample size, to compare among age classes and sexes, as it remains unclear whether vocal learning abilities depend on age and/or sex [14, 16]. Besides measurements of pulmonary capacity and airflow rate, behavioral data should be linked to the underlying neurobiology in this and other pinniped species [12, 30, 38, 45].

## Conclusion

We demonstrated, in a controlled experiment, a harbor seal’s ability for respiratory production learning, a proposed evolutionary precursor to vocal production learning. The seal not only mastered control over diverse durations of its vocalizations but also learned to manipulate sound duration in two distinct directions (short and long). Remarkably, the seal achieved durations that surpassed the pre-experimental range and produced vocalizations that covered durations of two orders of magnitude, showcasing an impressive level of vocal control and adaptability.

## Supplementary Information

Supplementary Material 1.

Supplementary Material 2.

## Data Availability

The research data used for this article has been uploaded in this submission.
