# Supplementary material for: A harbor seal (Phoca vitulina) shows extensive respiratory control in sound production
Source: BMC Ecol Evol. 2025 Sep 2;25:90. doi: 10.1186/s12862-025-02404-9 (PMC12403956; doi:10.1186/s12862-025-02404-9)
Supplement: Supplementary file 1 — Supplementary Material 1. [file 12862_2025_2404_MOESM1_ESM.docx]

**Supplementary Material**

**A harbor seal (*Phoca vitulina*) shows extensive respiratory control in sound production**

Diandra Duengen^1,2*^, Yannick Jadoul^1,4^, Andrea Ravignani^1,3,4,5*^

**Affiliations**

^1^*Comparative Bioacoustics Research Group, Max Planck Institute for Psycholinguistics, Nijmegen, The Netherlands*

^2^*Zoo Cleves (“Tiergarten Kleve”), Kleve, Germany*

^3^*Center for Music in the Brain, Department of Clinical Medicine, Aarhus University & The Royal Academy of Music, Aarhus, Denmark*

^4^*Department of Human Neurosciences, Sapienza University of Rome, Rome, Italy*

^5^*Research Center of Neuroscience "CRiN-Daniel Bovet", Sapienza University of Rome, Rome, Italy*


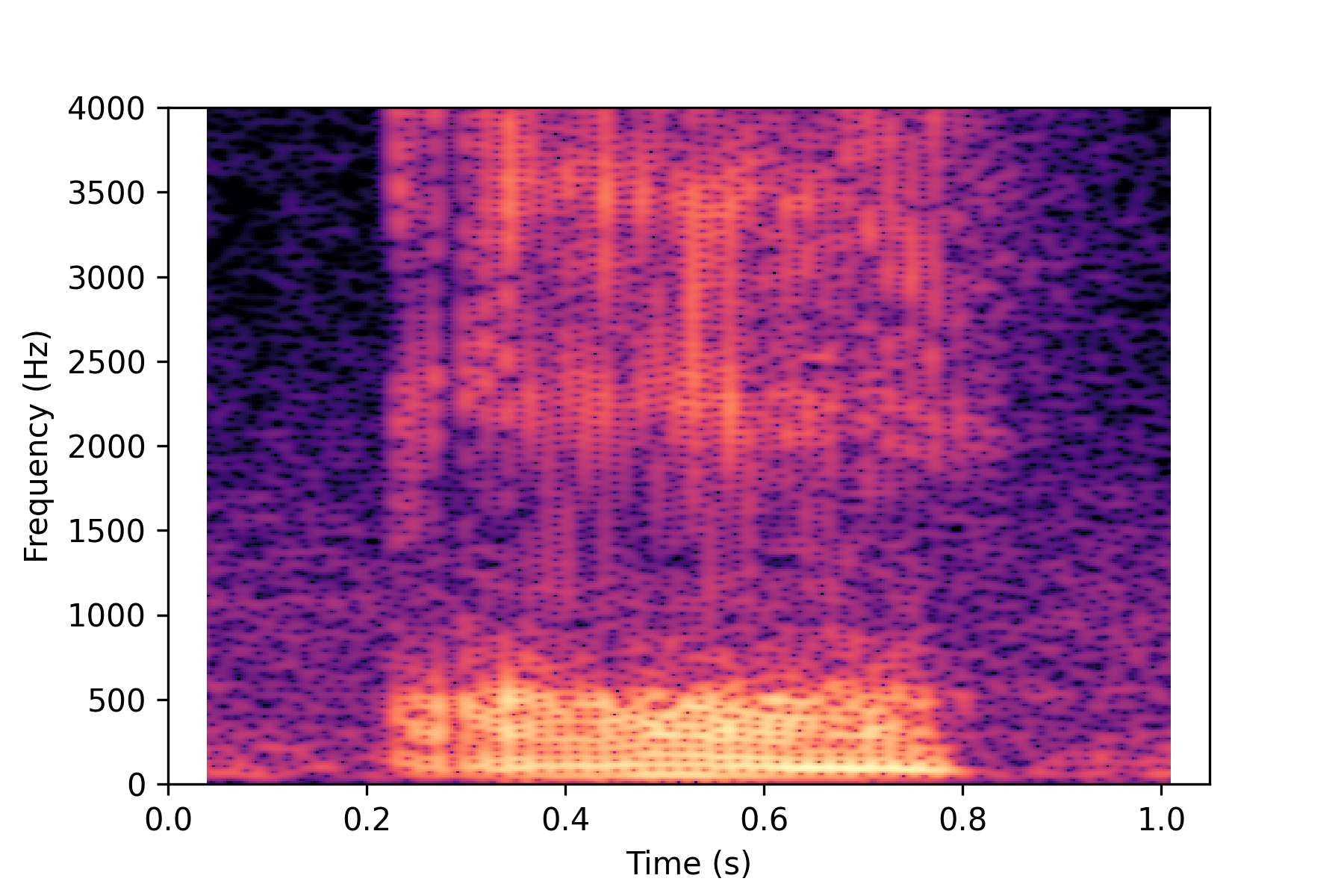


**Fig. S1:** Spectral representation of the seal’s focal vocalization. Spectrogram obtained with the Parselmouth package in Python [v0.4.3, Praat 6.1.38; window length = 0.04 s, dynamic range = 70 dB, max. frequency = 5 kHz; 1, 2, 3].


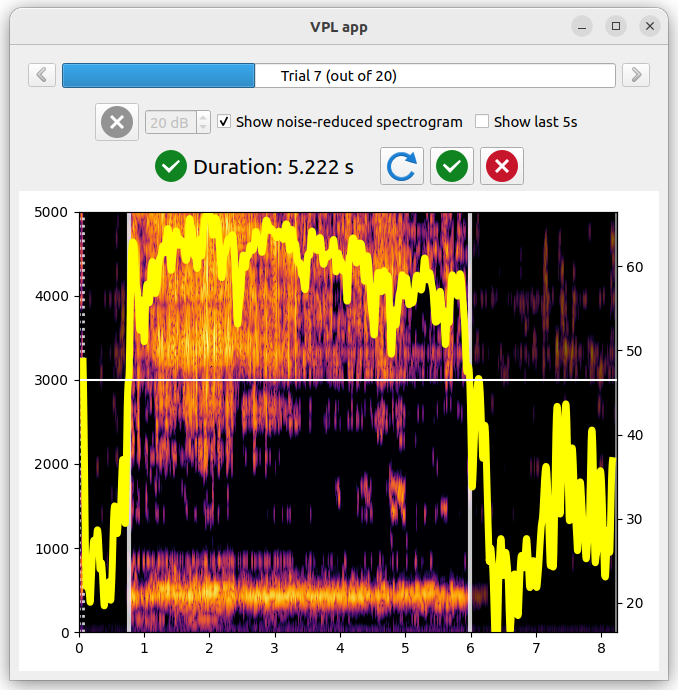


**Fig. S2**: Vocal learning application displaying the intensity-based detected vocalization and its calculated duration. The green checkmark to the left of the duration value depicts the automatically suggested response assessment (here: correct). Duration was directly displayed to the experimenter (except for the first five of sessions of shaping, where duration was inferred from the x-axis of the spectrogram).

**Tab. S1**: Descriptive statistical values for all vocalization’s durations in the different experimental sessions, grouped per short and long thresholds. Statistical test results refer to Mann-Whitney U values, comparing each threshold with the previous one. “Pre” data refer to the pre-experimental vocalizations’ duration distribution. Five sessions were excluded, which only had 10, 17, and 3 x 19 valid trials.

| **Session** | **Thres-hold (s)** | **Mean** | **Standard Deviation** | **Mini-mum** | **Maxi-mum** | **Median** | **Statistic** | **Significance Level** |
| --- | --- | --- | --- | --- | --- | --- | --- | --- |
| **Session_short_** | 0.611 | 0.323 | 0.177 | 0.149 | 1.027 | 0.274 | 28124.0 | p < 0.0001 |
|  | 0.272 | 0.320 | 0.174 | 0.087 | 0.870 | 0.250 | 4853.0 | p > 0.05 |
|  | 0.204 | 0.227 | 0.135 | 0.083 | 1.494 | 0.204 | 53025.5 | p < 0.0001 |
|  | 0.161 | 0.170 | 0.056 | 0.079 | 0.601 | 0.161 | 106410.5 | p < 0.0001 |
| **Pre** |  | **0.652** | **0.243** | **0.202** | **2.621** | **0.611** |  | |
| **Session_long_** | 0.611 | 0.729 | 0.182 | 0.259 | 1.200 | 0.726 | 43607.0 | p < 0.0001 |
|  | 0.768 | 0.943 | 0.273 | 0.525 | 2.044 | 0.879 | 6256.5 | p < 0.0001 |
|  | 1.092 | 1.281 | 0.347 | 0.368 | 2.123 | 1.295 | 3709.0 | p < 0.0001 |
|  | 1.443 | 1.836 | 0.365 | 1.110 | 2.792 | 1.808 | 1133.0 | p < 0.0001 |
|  | 1.905 | 2.185 | 0.538 | 0.549 | 3.498 | 2.191 | 2589.0 | p < 0.0001 |
|  | 2.446 | 3.357 | 0.667 | 1.834 | 5.078 | 3.358 | 444.0 | p < 0.0001 |
|  | 3.224 | 4.255 | 1.378 | 0.278 | 9.228 | 4.212 | 3529.0 | p < 0.0001 |
|  | 4.706 | 5.506 | 1.226 | 2.963 | 7.925 | 5.415 | 4215.0 | p < 0.0001 |


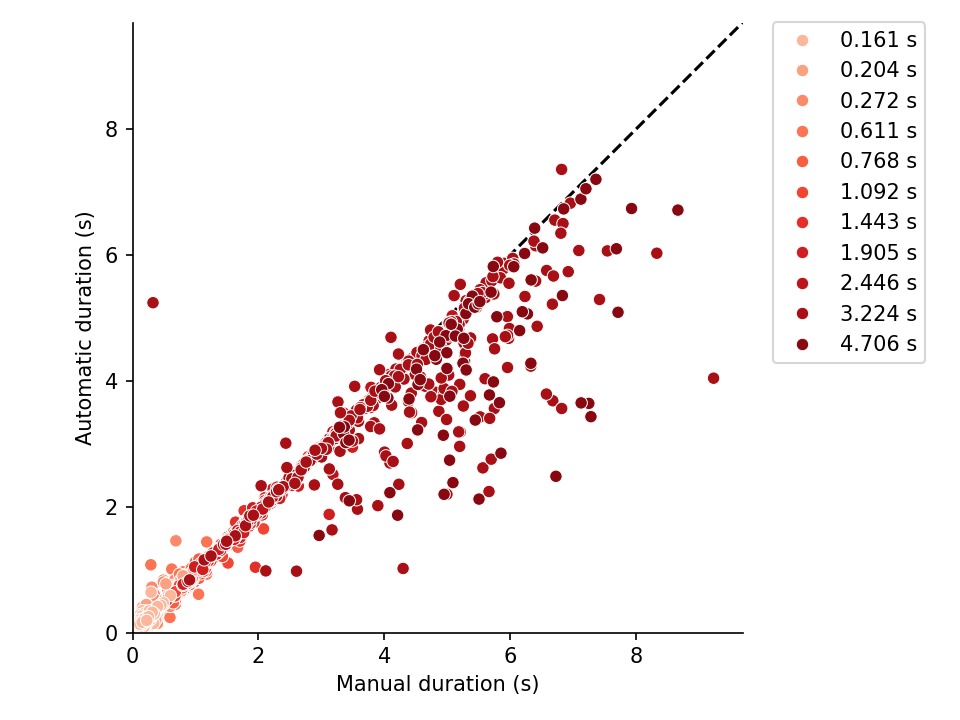
**Fig. S3:** A comparison of the application’s automatically detected vocalization’s durations (based on a recording’s intensity, see Method S1) with the duration of the manually annotated vocalizations. Spearman's rank correlation coefficient shows a strong and statistically significant correlation between the two duration measurements (statistic = 0.97695, p < 0.0001).

*Method S1: Automatic Detection of Vocal Responses*

The response vocalization in a recording was detected based on the recording’s intensity curve. By pressing a foot pedal at the start of a trial and releasing it after the end of the vocalization, the experimenter ensured that the only loud sound in the recording was the seal’s response. The application used Parselmouth [a development version based on Parselmouth v0.4.3, Praat v6.2.23; 1, 2, 3] to filter out background noise, calculate the intensity curve, and find the largest audio fragment where the intensity is more than 20 dB below the maximum intensity. For these steps the corresponding Praat functionality was “Sound: Reduce noise” (noise time range = 0 s – 0.25 s, default values for other parameters), “Sound: To Intensity” (minimum pitch = 50 Hz, time step = 0.001 s, default values for other parameters), and “Intensity: To TextGrid (silences)” (silence threshold = -20 dB, default values for other parameters). If several intervals were detected, e.g., if halfway through the vocalizations, the intensity curved dropped below the -20 dB threshold, the largest interval determined the measured duration of the vocalization.

Especially for short sessions, the experimenter’s manual assessment during and after the experiment ensured that this measurement did not underestimate the vocalization’s true duration. In all cases, suggested correct trials could be changed to incorrect but not vice versa. After the experiment, we manually verified the measured durations and results. All vocalizations were visually inspected using Praat v6.1.38 [1], manually annotated, and the duration calculated. This allowed us to compare the results to the application’s automatic measurements.

**References**

1. Boersma, P. and D. Weenink, *Praat: doing phonetics by computer* in *Glot International*. 2022.

2. Jadoul, Y., B. De Boer, and A. Ravignani, *Parselmouth for bioacoustics: automated acoustic analysis in Python.* Bioacoustics, 2023(33): p. 1-17.

3. Jadoul, Y., B. Thompson, and B. De Boer, *Introducing Parselmouth: A Python interface to Praat.* Journal of Phonetics, 2018. **71**: p. 1-15.
