# Supplementary material for: A harbor seal (Phoca vitulina) shows extensive respiratory control in sound production
Source: BMC Ecol Evol. 2025 Sep 2;25:90. doi: 10.1186/s12862-025-02404-9 (PMC12403956; doi:10.1186/s12862-025-02404-9)
Supplement: Supplementary file 2 — Supplementary Material 2. [file 12862_2025_2404_MOESM2_ESM.zip › Analysis.html]

Analysis


In [1]:

```
import numpy as np
import pandas as pd
import scipy.stats
```

# Data and pre-processing¶

In [2]:

```
# Pre-experimental vocalizations
pre_experimental_durations_df = pd.read_csv("pre_experimental_durations.csv")

# Experiment results
experiment_log_df = pd.read_csv("experiment_log.csv")
experiment_durations_df = pd.read_csv("experiment_durations.csv")
experiment_durations_manual_df = pd.read_csv("experiment_durations_manual.csv")
```

In [3]:

```
full_sessions = experiment_log_df.loc[lambda df: df['valid'] == 20, 'session']

# Filter out invalid sessions, repeated trials, and trials with multiple responses, and merge with experiment metadata
experiment_durations_manual_df = experiment_durations_manual_df[lambda df: df['session'].isin(full_sessions) & ~df['result'].isna() & (df['manual_annotation'] == 'v')]
experiment_durations_manual_df = pd.merge(experiment_durations_manual_df, experiment_log_df, how='inner', on=['session'], validate='many_to_one')

# The 'duration' column of `experiment_durations_manual_df` already contains the corresonding values from `experiment_durations_df`
experiment_durations_manual_df = experiment_durations_manual_df.rename(columns={'duration': 'auto_duration'})
```

In [4]:

```
thresholds_short = sorted(experiment_log_df.loc[lambda df: df['direction'] == 'short', 'threshold'].unique(), reverse=True)
thresholds_long = sorted(experiment_log_df.loc[lambda df: df['direction'] == 'long', 'threshold'].unique())
```

# Descriptive statistics¶

In [5]:

```
def get_descriptive_stats(values):
    return {
        "Mean": np.mean(values),
        "Std Dev": np.std(values),
        "Minimum": np.min(values),
        "Maximum": np.max(values),
        "Median": np.median(values)
    }


rows = []
rows.append(pd.Series(get_descriptive_stats(pre_experimental_durations_df['duration']), name="Pre-experimental"))

for threshold in thresholds_short:
    threshold_durations_df = experiment_durations_manual_df.loc[lambda df: (df['direction'] == 'short') & (df['threshold'] == threshold)]
    rows.append(pd.Series(get_descriptive_stats(threshold_durations_df['manual_duration']), name=f"Short, {threshold}"))

for threshold in thresholds_long:
    threshold_durations_df = experiment_durations_manual_df.loc[lambda df: (df['direction'] == 'long') & (df['threshold'] == threshold)]
    rows.append(pd.Series(get_descriptive_stats(threshold_durations_df['manual_duration']), name=f"Long, {threshold}"))

descriptive_stats_df = pd.DataFrame(rows)
descriptive_stats_df
```

Out[5]:

|  | Mean | Std Dev | Minimum | Maximum | Median |
| --- | --- | --- | --- | --- | --- |
| Pre-experimental | 0.652145 | 0.243070 | 0.201788 | 2.620980 | 0.611373 |
| Short, 0.611 | 0.322835 | 0.177138 | 0.148843 | 1.027447 | 0.273872 |
| Short, 0.272 | 0.320153 | 0.173858 | 0.087035 | 0.869949 | 0.249513 |
| Short, 0.204 | 0.226856 | 0.135351 | 0.082790 | 1.494468 | 0.203791 |
| Short, 0.161 | 0.170487 | 0.056008 | 0.079252 | 0.601467 | 0.160627 |
| Long, 0.611 | 0.728558 | 0.182023 | 0.258984 | 1.200103 | 0.726006 |
| Long, 0.768 | 0.942658 | 0.272953 | 0.524819 | 2.043572 | 0.878849 |
| Long, 1.092 | 1.281407 | 0.346530 | 0.367956 | 2.122824 | 1.295085 |
| Long, 1.443 | 1.836372 | 0.365203 | 1.110159 | 2.791628 | 1.808066 |
| Long, 1.905 | 2.184905 | 0.538381 | 0.549104 | 3.498413 | 2.190754 |
| Long, 2.446 | 3.356609 | 0.667017 | 1.834120 | 5.077794 | 3.357599 |
| Long, 3.224 | 4.254631 | 1.378033 | 0.277540 | 9.227959 | 4.212390 |
| Long, 4.706 | 5.505627 | 1.225862 | 2.963462 | 7.925208 | 5.415122 |

# Statistical difference between successive thresholds¶

In [6]:

```
scipy.stats.normaltest(pre_experimental_durations_df['duration'])
```

Out[6]:

```
NormaltestResult(statistic=np.float64(484.5940501630156), pvalue=np.float64(5.912062621507615e-106))
```

Pre-experimental durations are not normally distributed, so we continue with non-parametric tests.

In [7]:

```
rows = []

threshold = thresholds_short[0]
threshold_durations_df = experiment_durations_manual_df.loc[lambda df: (df['direction'] == 'short') & (df['threshold'] == threshold)]
result = scipy.stats.mannwhitneyu(pre_experimental_durations_df['duration'], threshold_durations_df['manual_duration'])
rows.append(pd.Series({'Statistic': result.statistic, 'p-value': result.pvalue}, name=f"Pre-experimental vs. Short, {threshold}"))

for prev_threshold, threshold in zip(thresholds_short[:-1], thresholds_short[1:]):
    prev_threshold_durations_df = experiment_durations_manual_df.loc[lambda df: (df['direction'] == 'short') & (df['threshold'] == prev_threshold)]
    threshold_durations_df = experiment_durations_manual_df.loc[lambda df: (df['direction'] == 'short') & (df['threshold'] == threshold)]
    result = scipy.stats.mannwhitneyu(prev_threshold_durations_df['manual_duration'], threshold_durations_df['manual_duration'])
    rows.append(pd.Series({
        'Statistic': result.statistic,
        'p-value': result.pvalue
    }, name=f"Short, {prev_threshold} vs. Short, {threshold}"))

threshold = thresholds_long[0]
threshold_durations_df = experiment_durations_manual_df.loc[lambda df: (df['direction'] == 'long') & (df['threshold'] == threshold)]
result = scipy.stats.mannwhitneyu(pre_experimental_durations_df['duration'], threshold_durations_df['manual_duration'])
rows.append(pd.Series({'Statistic': result.statistic, 'p-value': result.pvalue}, name=f"Pre-experimental vs. Long, {threshold}"))

for prev_threshold, threshold in zip(thresholds_long[:-1], thresholds_long[1:]):
    prev_threshold_durations_df = experiment_durations_manual_df.loc[lambda df: (df['direction'] == 'long') & (df['threshold'] == prev_threshold)]
    threshold_durations_df = experiment_durations_manual_df.loc[lambda df: (df['direction'] == 'long') & (df['threshold'] == threshold)]
    result = scipy.stats.mannwhitneyu(prev_threshold_durations_df['manual_duration'], threshold_durations_df['manual_duration'])
    rows.append(pd.Series({
        'Statistic': result.statistic,
        'p-value': result.pvalue
    }, name=f"Long, {prev_threshold} vs. Long, {threshold}"))

successive_thresholds_df = pd.DataFrame(rows)
successive_thresholds_df
```

Out[7]:

|  | Statistic | p-value |
| --- | --- | --- |
| Pre-experimental vs. Short, 0.611 | 28124.0 | 1.135871e-17 |
| Short, 0.611 vs. Short, 0.272 | 4853.0 | 3.661018e-01 |
| Short, 0.272 vs. Short, 0.204 | 50325.5 | 2.526730e-20 |
| Short, 0.204 vs. Short, 0.161 | 106410.5 | 1.729746e-26 |
| Pre-experimental vs. Long, 0.611 | 43607.0 | 6.172390e-10 |
| Long, 0.611 vs. Long, 0.768 | 6256.5 | 1.178512e-13 |
| Long, 0.768 vs. Long, 1.092 | 3709.0 | 9.609157e-16 |
| Long, 1.092 vs. Long, 1.443 | 1133.0 | 2.836584e-17 |
| Long, 1.443 vs. Long, 1.905 | 2589.0 | 8.733512e-07 |
| Long, 1.905 vs. Long, 2.446 | 444.0 | 1.555964e-14 |
| Long, 2.446 vs. Long, 3.224 | 3529.0 | 9.239870e-06 |
| Long, 3.224 vs. Long, 4.706 | 4215.0 | 5.808643e-09 |

# Statistical difference between pre-experimental and the final sessions¶

In [8]:

```
rows = []

final_session_short = experiment_durations_manual_df.loc[lambda df: df['direction'] == 'short', 'session'].max()
final_session_durations_df = experiment_durations_manual_df.loc[lambda df: df['session'] == final_session_short]
threshold = final_session_durations_df['threshold'].unique()[0]
result = scipy.stats.mannwhitneyu(pre_experimental_durations_df['duration'], final_session_durations_df['manual_duration'])
rows.append(pd.Series({
    'Mean': np.mean(final_session_durations_df['manual_duration']),
    'Std Dev': np.std(final_session_durations_df['manual_duration']),
    'Statistic': result.statistic,
    'p-value': result.pvalue
}, name=f"Pre-experimental vs. Final short session ({threshold})"))

final_session_long = experiment_durations_manual_df.loc[lambda df: df['direction'] == 'long', 'session'].max()
final_session_durations_df = experiment_durations_manual_df.loc[lambda df: df['session'] == final_session_long]
threshold = final_session_durations_df['threshold'].unique()[0]
result = scipy.stats.mannwhitneyu(pre_experimental_durations_df['duration'], final_session_durations_df['manual_duration'])
rows.append(pd.Series({
    'Mean': np.mean(final_session_durations_df['manual_duration']),
    'Std Dev': np.std(final_session_durations_df['manual_duration']),
    'Statistic': result.statistic,
    'p-value': result.pvalue
}, name=f"Pre-experimental vs. Final long session ({threshold})"))

final_sessions_df = pd.DataFrame(rows)
final_sessions_df
```

Out[8]:

|  | Mean | Std Dev | Statistic | p-value |
| --- | --- | --- | --- | --- |
| Pre-experimental vs. Final short session (0.161) | 0.159696 | 0.041538 | 15443.0 | 9.029992e-14 |
| Pre-experimental vs. Final long session (4.706) | 5.335947 | 1.312403 | 0.0 | 1.626986e-12 |

# Comparison between manual and automatic durations¶

In [9]:

```
scipy.stats.normaltest(experiment_durations_manual_df['manual_duration'])
```

Out[9]:

```
NormaltestResult(statistic=np.float64(587.5454649231981), pvalue=np.float64(2.606893914692659e-128))
```

In [10]:

```
scipy.stats.normaltest(experiment_durations_manual_df['auto_duration'])
```

Out[10]:

```
NormaltestResult(statistic=np.float64(549.0081490403605), pvalue=np.float64(6.0868861108207906e-120))
```

Neither the automatically extracted durations nor the manually annotated durations are normally distributed, so we again use a non-parametric test.

In [11]:

```
scipy.stats.spearmanr(experiment_durations_manual_df['manual_duration'], experiment_durations_manual_df['auto_duration'])
```

Out[11]:

```
SignificanceResult(statistic=np.float64(0.9762648777976944), pvalue=np.float64(0.0))
```
